# Four new species of *Dolichopoda* Bolivar, 1880 from Southern Sporades and Western Turkey (Orthoptera, Rhaphidophoridae, Dolichopodainae)

**DOI:** 10.3897/zookeys.201.2609

**Published:** 2012-06-14

**Authors:** Mauro Rampini, Claudio Di Russo, Mehmet Sait Taylan, Arianna Gelosa, Marina Cobolli

**Affiliations:** 1 Dipartimento di Biologia e Biotecnologie ”C. Darwin” (Zoologia), Università degli Studi di Roma “La Sapienza”, Viale dell’Università 32, 00185 Roma, Italy; 2 Biology Department, Science Institute, Akdeniz University Dumlupınar Boulevard Post Code: 07058 Campus Antalya, Turkey; 3 Via Accademia degli Agiati 120, 00147 Roma, Italy

**Keywords:** Cave crickets, *Dolichopoda*, new species, Eastern Aegean, Western Turkey

## Abstract

A description of four new species of *Dolichopoda* Bolivar, 1880 (Orthoptera, Rhaphidophoridae) from Eastern Aegean region (Southern Sporades), including Western Turkey, is reported. This brings to a total of 11 the number of *Dolichopoda* species recorded for caves of the Aegean area. Overall, these species show a high degree of morphological homogeneity and they are very close to *Dolichopoda paraskevi* Boudou-Saltet, 1973 from Crete and *Dolichopoda naxia* Boudou-Saltet, 1972 from Cyclades (Naxos Island). The Western Turkish species are morphologically not closely related to the other Anatolian species; this suggests an independent origin for the taxa occurring in the Southern Taurus and Black Sea regions. These new data help to better define the already high level of diversity of the Hellenic *Dolichopoda* and strengthen the hypothesis that the central area of dispersal for the genus would correspond to the ancient Aegean plate.

## Introduction

The subfamily Dolichopodainae Brunner von Wattenwyl, 1888 (Orthoptera, Rhaphidophoridae) is limited to the Northern hemisphere; several species belonging to the genus *Dolichopoda* Bolivar, 1880 inhabit caves of Southern Europe and Asia Minor. To date the genus includes a total 48 species (Heller et al. 1998, Otte 2000, Eades et al. 2012). The genus *Dolichopoda* is mainly widespread in the Mediterranean area from the Pyrenees to the Caucasus (Baccetti 1982, Di Russo and Sbordoni 1998); its diversity in terms of species richness peaks in Southern and South Eastern Europe (Mediterranean basin, Apennines, Balkan and Peloponnesus). In particular, 25 of the known species have been reported for many caves in continental and insular Greece (Boudou-Saltet 1983, Willemse 1984, Rampini et al. 2008). Among these, seven species only are found on Aegean Islands: *Dolichopoda naxia* Boudou-Saltet, 1972 from Naxos, *Dolichopoda thasosensis* Chopard, 1964 from Thasos, *Dolichopoda cassagnaui* Boudou-Saltet, 1971, *Dolichopoda makrykapa* Boudou-Saltet, 1980 and *Dolichopoda ochtoniai* Boudou-Saltet, 1983 from Eubea, *Dolichopoda saraolakosi* Boudou-Saltet, 1983 from Skyros and *Dolichopoda paraskevi* Boudou-Saltet, 1973 from Crete. On the other hand, until now only five species of this genus were recorded for Turkey (Bolivar 1899, Di Russo and Rampini 2006, Galvagni 2006, Di Russo et al. 2007, Rampini and Di Russo 2008, Taylan et al. 2011, Ünal 2011). Two of these (*Dolichopoda sbordonii* Di Russo and Rampini, 2006 and *Dolichopoda lycia* (Galvagni, 2006)) are limited to caves around Antalya, while other two (*Dolichopoda aranea* Bolivar, 1899 and *Dolichopoda pusilla* Bolivar, 1899) are restricted to a few localities of the Eastern Taurus and only *Dolichopoda noctivaga* Di Russo and Rampini, 2007 is widespread in caves and epigean habitats in Northern Turkey throughout the Black Sea region (Taylan et al. 2011).

In recent years several *Dolichopoda* were collected by us for the first time in caves of the Southern Sporades and Aegean Turkey. At a first examination these new populations did not show remarkable morphological differences resulting all very similar and close to one other. However, a genetic study based on mitochondrial DNA (mtDNA) revealed a noticeable level of genetic divergence, comparable to that usually found among morphologically divergent species of the genus (Allegrucci et al. 2009, Taylan et al. 2011). These interesting results prompted us to carry out a more detailed morphological survey of these taxa. This allowed us to describe four new *Dolichopoda* species from the Eastern Agean: two from caves on Samos, one on Kalymnos and one from the Aegean Turkey (İzmir and Aydın Province).

All these new species are attributable to the sub-genus *Dolichopoda* s. str. (Baccetti 1958), because of the absence of spines on all femora, the occurrence of spines on the fore tibia and a non-bifurcate epiphallus.

The material was brought together by M.S. Taylan, M. Rampini and C. Di Russo for collective study and deposition in: Museum of Zoology, University of Rome “La Sapienza” (MZUR); Akdeniz University, Science Institute, Biology Department, Antalya, Turkey (AUZM). The localities of the studied species are presented on the distribution map shown in Figure 22.

## Systematics

### Dolichopoda (Dolichopoda) sutini

Rampini & Taylan sp. n.

urn:lsid:zoobank.org:act:92E36710-6542-4B84-A645-F34534D3D40E

http://species-id.net/wiki/Dolichopoda_sutini

Figures 1–5

#### Type-locality.

The Sütini cave is situated on the road from Selçuk to Sirince (İzmir Province, Turkey).

#### Material examined.

Thirty-three specimens.

#### Type material.

Holotype male. Turkey, İzmir, Selçuk, Sütini cave, 27.06.2008, M.S. Taylan leg. (AUZM).

Paratypes: 5 males, 5 females, 1 nymph, same data and collector as for holotype; 1 male,12 females, 8 nymphs, Turkey, Aydın, Söke, Aşıkali cave, 13.07.2009, M.S. Taylan leg. (AUZM).

#### Differential diagnosis.

The size is relatively small with the hind legs strongly elongated. This species is close to Aegean species *Dolichopoda naxia* from Naxos, and *Dolichopoda paraskevi* from Crete but differs from them for the number of spines on the hind tibia (19); these are 25 in *Dolichopoda naxia* and 16 in *Dolichopoda paraskevi*. The tenth tergite differs from that of *Dolichopoda naxia* and *Dolichopoda paraskevi* for the shape and size of the lateral lobes and for the deeper medial incision. The male subgenital plate appears close to that of *Dolichopoda paraskevi* while it differs from *Dolichopoda naxia*; here the lateral lobes are trapezoidal. The median process of the epiphallus, flattened and rather enlarged at the base, is very close to that of *Dolichopoda paraskevi*, while in *Dolichopoda naxia* it is narrow and more elongated. From a lateral view the median process differs from that of the other two species being more curved. The female subgenital plate, with rounded lobes, is similar to that of *Dolichopoda paraskevi* but differs from *Dolichopoda naxia* for the deeper medial incision between lobes. The ovipositor, similar in length and shape, is different only for the number of denticles on the inferior valves.

#### Description.

**Figures 1–5.**
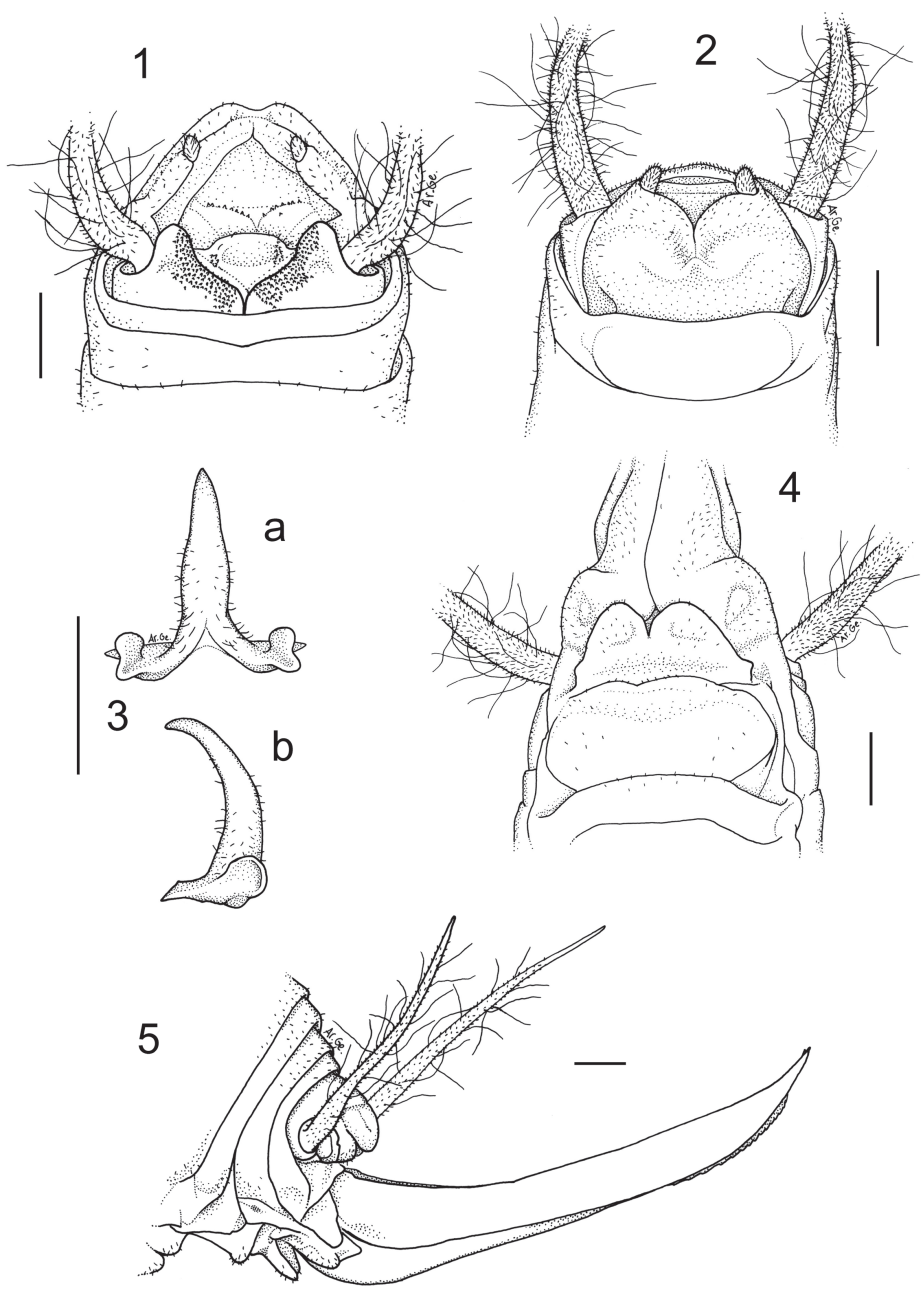
*Dolichopoda sutini* sp. n. Holotype male, **1** X tergite, dorsal view **2** subgenital plate, ventral view **3** epiphallus: a- dorsal view, b- lateral view. Female **4** subgenital plate, ventral view **5** ovipositor, lateral view. Scale bars: 1 mm.

Male (holotype)**.** Body colour pale-testaceous, uniform with the exception of the posterior margins of the tergites, which are darker. Legs long, slender and yellow-testaceous in colour with the femora unarmed. Fore tibia armed with 4/5 spines on both sides of the inferior edge and a pair of spurs of equal length on the apex. Mid tibia with 3/4 short spines on both sides of the upper edge, 4/5 spines on the lower edge and two apical spurs similar to those of the fore tibia. The hind tibia is longer with 18/19 spines of varying length on both sides of the upper edge and 1/3 homogeneous spines on the lower edge. Tenth tergite on the posterior edge with two prominent lateral lobes triangular in shape with rounded apex separated from one another by a deep median incision (Figure 1). Subgenital plate globular at the bottom, with a deep median incision that runs for half of the total length; the symmetrical lateral lobes, triangular in shape, hold two evident styli cylindrical in shape (Figure 2). The epiphallus is sclerotized and shows a median process relatively long, almost flattened and with an acute apex; it appears large at the base and without lateral constrictions; from the side, the median process is uniformly thickened and curved; the basal processes are rather developed and slightly divergent (Figure 3 a, b). The accessory apparatus is sclerotized and composed by an uneven Y shaped piece, showing strong spines at the base, and by even partially trapezoidal valves.

Length(mm): body 14.0; pronotum 3.0; fore femora 16.2; middle femora 16.5; hind femora 22.4; fore tibia 16.8; middle tibia 17.3; hind tibia 30.2; hind tarsus 10.2; 1st article of hind tarsus 5.0.

Female**.** The length of the body ranges between 14.8–15.9 mm (ovipositor excluded) and the general form of the female is similar to the male. The subgenital plate is triangular (Figure 4), with two prominent lobes deeply incised and rounded at the posterior edges. The ovipositor has an average length of 10 mm, rather enlarged at the base and little curved on the superior edge (Figure 5).

The superior valves have a pointed apex and curves upwards, whereas the inferior valves are a little shorter than the superior ones, are rounded at the apex and have 16–17 denticles.

#### Etymology.

The new species name refers to the Sütini cave in Selçuk (İzmir Province).

### Dolichopoda (Dolichopoda) giulianae

Rampini & Di Russo sp. n.

urn:lsid:zoobank.org:act:CAEFB8D0-2038-48CE-912B-F8FBDA87CB85

http://species-id.net/wiki/Dolichopoda_giulianae

Figures 6–10

#### Type-locality.

In the North-West part of Pythagorion (Samos Island) is located the monastery of Panaghia Spiliani; here, 95 steps lead down into a big cave with a church that is dedicated to the Virgin Mary. The caves was originally used to extract blocks of massive limestone to build walls and many buildings for the town of Samos.

#### Material examined.

Sixteen specimens.

#### Type material.

Holotype male, Greece, Samos Isl., Pythagorion,Panaghia Spiliani cave, 04.04.2008, M. Rampini, C. Di Russo leg. (MZUR).

Paratypes: 4 males, 2 females, 3 nymphs, same locality date and collectors as holotype (MZUR). 1 female, 5 nymphs, same locality as holotype 21.08.2002, F. Gasparo leg. (MZUR).

#### Differential diagnosis.

The overall appearance of this new species is very similar to that *Dolichopoda sutini* from İzmir. Differences are found in the squared and divergent lobes of the tenth male tergite, in the shape of the epiphallus (less enlarged at the base) and in the shorter styli of the subgenital plate. The female subgenital plate is similar to that of *Dolichopoda paraskevi* but differs from *Dolichopoda sutini* for the more rounded lobes. The ovipositor has fewer apex denticles on the inferior valves.

#### Description.

**Figures 6–10.**
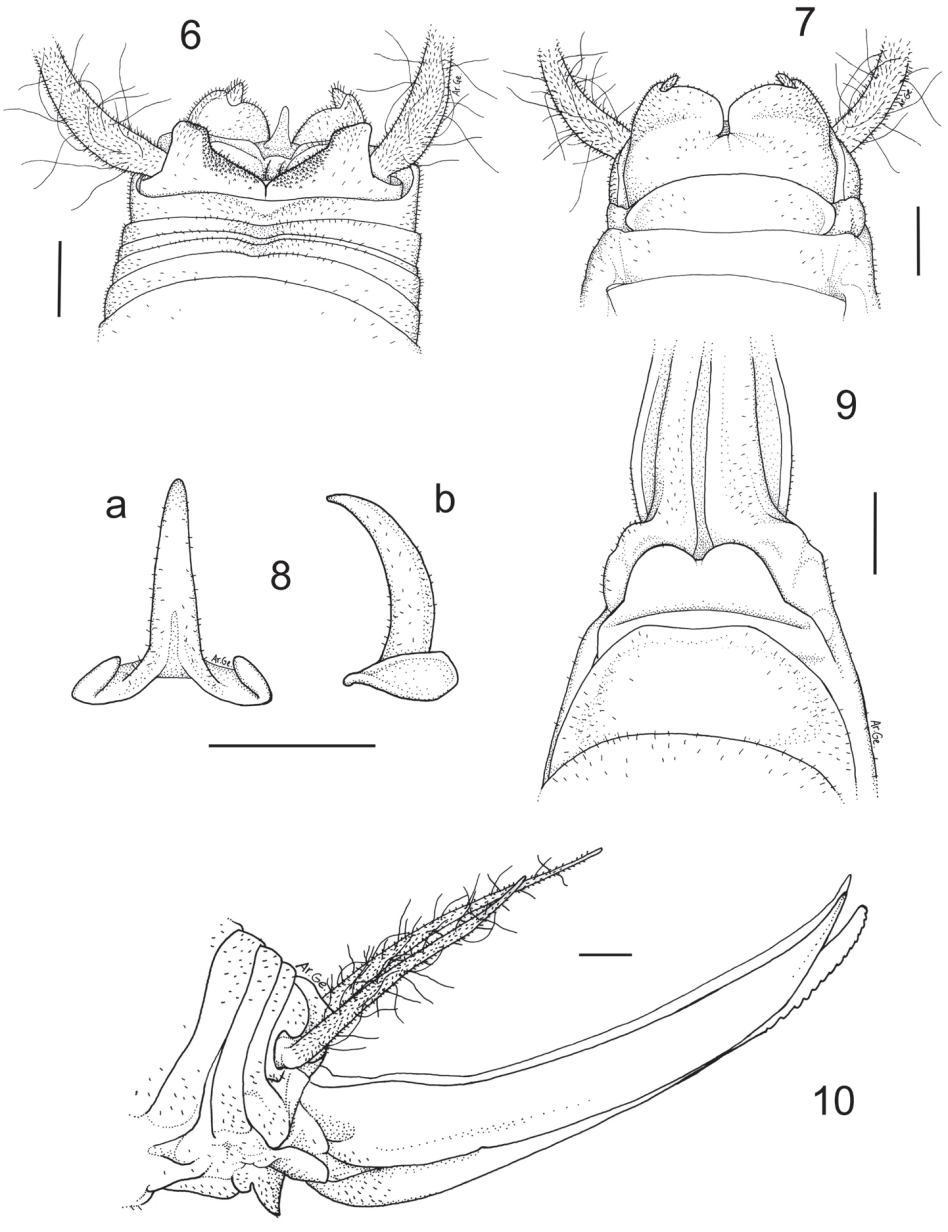
*Dolichopoda giulianae* sp. n. Holotype male **6** X tergite, dorsal view **7** subgenital plate, ventral view **8** epiphallus, **a** dorsal view **b** lateral view. Female **9** subgenital plate, ventral view **10** ovipositor, lateral view. Scale bars: 1 mm.

Male (holotype). Size relatively small. Body and appendages coloration as in the previous species. Femora unarmed. Fore tibia armed with 3 spines on both sides of the upper edge and 3/5 spines on the lower edges. Mid tibia with 5 short spines on both sides of the upper edge, 4 spines on the lower edge. The hind tibia is longer with 19/22 spines of varying length on both sides of the upper edge and 0/2 homogeneous spines on the lower edge. The tenth tergite shows two prominent lobes on the posterior edge, almost squared at the apex (Figure 6). The subgenital plate, globular at the bottom, is similar to that of *Dolichopoda sutini*. The lateral lobes, triangular in shape, hold two short conical styli (Figure 7). The epiphallus is sclerotized and shows a long flattened median process, acute at the apex; from the side, it appears uniformly curved (Figure 8 a, b). The accessory apparatus looks similar to that of *Dolichopoda sutini*.

Lenght (mm): body 14.5; pronotum 3.5; fore femora 14.0; middle femora 14.0; hind femora 22.0; fore tibia 15.5; middle tibia 16.5; hind tibia 28.0; hind tarsus 11.0; 1st segment of hind tarsus 5.5.

Female. General appearance as in the male. The length of the body ranges between 15.0 and 18.0 mm (ovipositor excluded). The subgenital plate is trapezoidal with two rounded lobes (Figure 9). The ovipositor has an average length of 11.0 mm, is rather enlarged at the base and is regularly curved on the superior edge (Figure 10); the apex is pointed and curved upwards. The inferior valves have 15 apical denticles.

#### Etymology.

The new species is dedicated to our colleague and friend Giuliana Allegrucci for her useful and active collaboration in this study.

### Dolichopoda (Dolichopoda) kalithea

Di Russo & Rampini sp. n.

urn:lsid:zoobank.org:act:E82231B2-8FF8-4ED5-BE7D-59B803BEBDDF

http://species-id.net/wiki/Dolichopoda_kalithea

Figures 11–15

#### Type-locality.

The cave, with a chapel inside, is located East of Kalithea village in the Western part of Mount Kerkis, inside the canyon Kakoperato, which starts from a little monastery called Panaghias Kakoperato. The cave is known for the presence of the endemic Staphylinid rove beetle *Tychobythinus brachati* Besuchet, 2008.

#### Material examined.

Sixteen specimens.

#### Type material.

Holotype male, Greece, Samos Isl., Mount Kerkis, Kakoperato canyon, 660 m, Kakoperato cave (or Trypa Tse-Tse cave), 05.04.2008, C. Di Russo, M. Rampini leg.

Paratypes: 6 males, 1 male, 2 nymphs, same locality, date and collectors (MZUR). South-Eastern slopes of Mount Kerkis, Marathokampos, 320 m, Sarandaskaliotissa cave (near Pythagoras cave), 1 male, 2 females, 3 nymphs, 05.04.2008, C. Di Russo, M. Rampini leg. (MZUR).

#### Differential diagnosis.

Shape and coloration as in the previous species. Size relatively large, with very long legs. This species, owing to the triangular lobes of the tenth tergite (almost fully separated by a large concavity) and to the trapezoidal lobes of the subgenital plate is close to *Dolichopoda naxia*. On the contrary the pronounced curve of the median process of the epiphallus differs markedly from that of *Dolichopoda naxia* while it resembles that of *Dolichopoda sutini* and of *Dolichopoda giulianae*. The female subgenital plate differs from that of both *Dolichopoda sutini* and *Dolichopoda giulianae* for the lower incision between the two lobes.

#### Description. 

**Figures 11–15.**
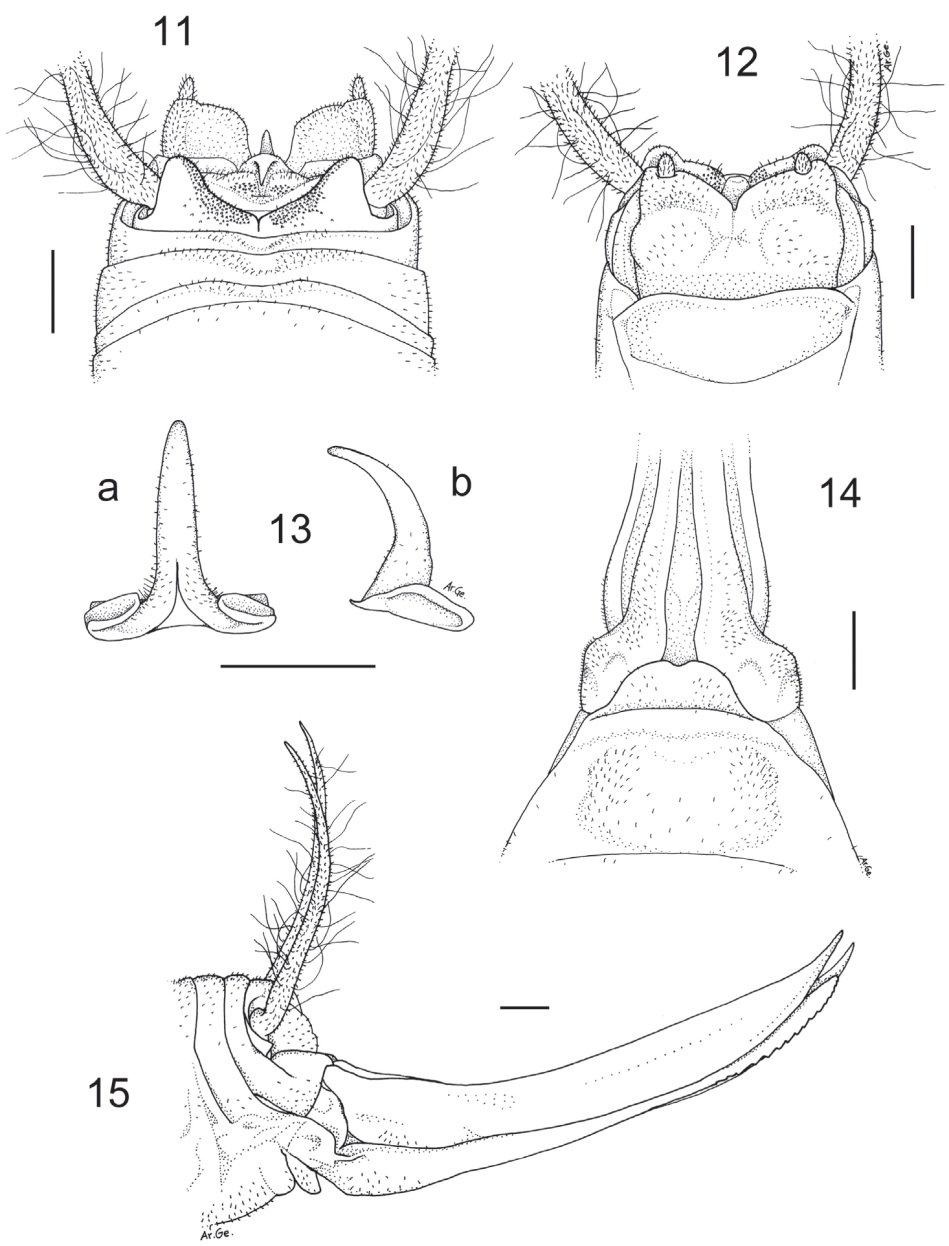
*Dolichopoda kalithea* sp. n. Holotype male, **11** X tergite, dorsal view **12** subgenital plate, ventral view **13** epiphallus **a** dorsal view **b** lateral view. Female **14** subgenital plate, ventral view **15** ovipositor, lateral view. Scale bars: 1 mm.

Male (holotype). Body and appendages coloration as in the previous species. Femora unarmed. Fore tibia armed with 1/4 spines on both sides of the upper edge and 3/5 spines on the lower edges. Mid tibia with 1/4 short spines on both sides of the upper edge, 4 spines on the lower edge. The hind tibia is longer with 13/20 spines of varying length on both sides of the upper edge and 0/3 homogeneous spines on the lower edge. The tenth tergite has two triangular lobes quite developed and separated by a large concavity (Figure 11). The subgenital plate shows two trapezoidal lobes, straight on the posterior edges and separated by a relatively short incision (Figure 12); the lobes hold two prominent cylindrical styli. The epiphallus is sclerotized and shows a quite flattened median process with an enlarged base; laterally, it appears rather thick at the base and strongly arched distally (Figure 13). The accessory apparatus is similar to that of the previous species.

Length(mm): body 16.0; pronotum 3.0; fore femora 13.0; middle femora 14.0; hind femora 21.5; fore tibia 15.5; middle tibia 16.5; hind tibia 27.0; hind tarsus 10.5; 1st article of hind tarsus 5.0.

Female. General appearance as in the male. The length of the body ranges between 19.0 and 21.0 mm (ovipositor excluded). The subgenital plate is rounded and slightly incised in the middle (Figure 14). The ovipositor has an average length of 12.0 mm, rather enlarged at the base and regularly curved on the superior edge (Figure 15). The superior valves have a pointed apex and curves upwards, the inferior valves have 14 denticles .

#### Etymology.

The new species takes its name from the Kalithea village.

### Dolichopoda (Dolichopoda) calidnae

Rampini & Di Russo sp. n.

urn:lsid:zoobank.org:act:58C66299-D839-4EEC-852C-D11AC9E3E78F

http://species-id.net/wiki/Dolichopoda_calidnae

Figures 16–21

#### Type-locality.

Foot of the Mt. Flaska, in the cave of the Nymphs**,** also called the cave of the Seven Virgins.

#### Material examined.

Fifteen specimens.

#### Type material.

Holotype male, Greece, Kalymnos Isl., Pothia, Seven Virgins cave (Sanctuary of the Nimphs or Epta Parthenes cave), 28.03.2004, M. Rampini, C. Di Russo leg.

Paratypes: 1 male, 5 females, same data and collectors; Skalia village, Skalia cave (Mts Flaska), 3 males, 5 nymphs, 28.03.2004, M. Rampini, C. Di Russo leg. (MZUR).

#### Differential diagnosis.

Colour of the body uniformly pale-testaceous, legs more yellowish. The male tenth tergite shows expanded lateral lobes of triangular shape with an acute apex. The subgenital plate, with trapezoidal lobes, is similar to that of *Dolichopoda naxia* but with short apical styli on the posterior edge. The epiphallus is long and slender as in *Dolichopoda naxia*, with a little curved median process, stretched and narrower than in the previous species. The female subgenital plate is morphologically different from that of all the other species showing a triangular shape with two moderately incised lobes in the middle.

#### Description.

**Figures 16–20.**
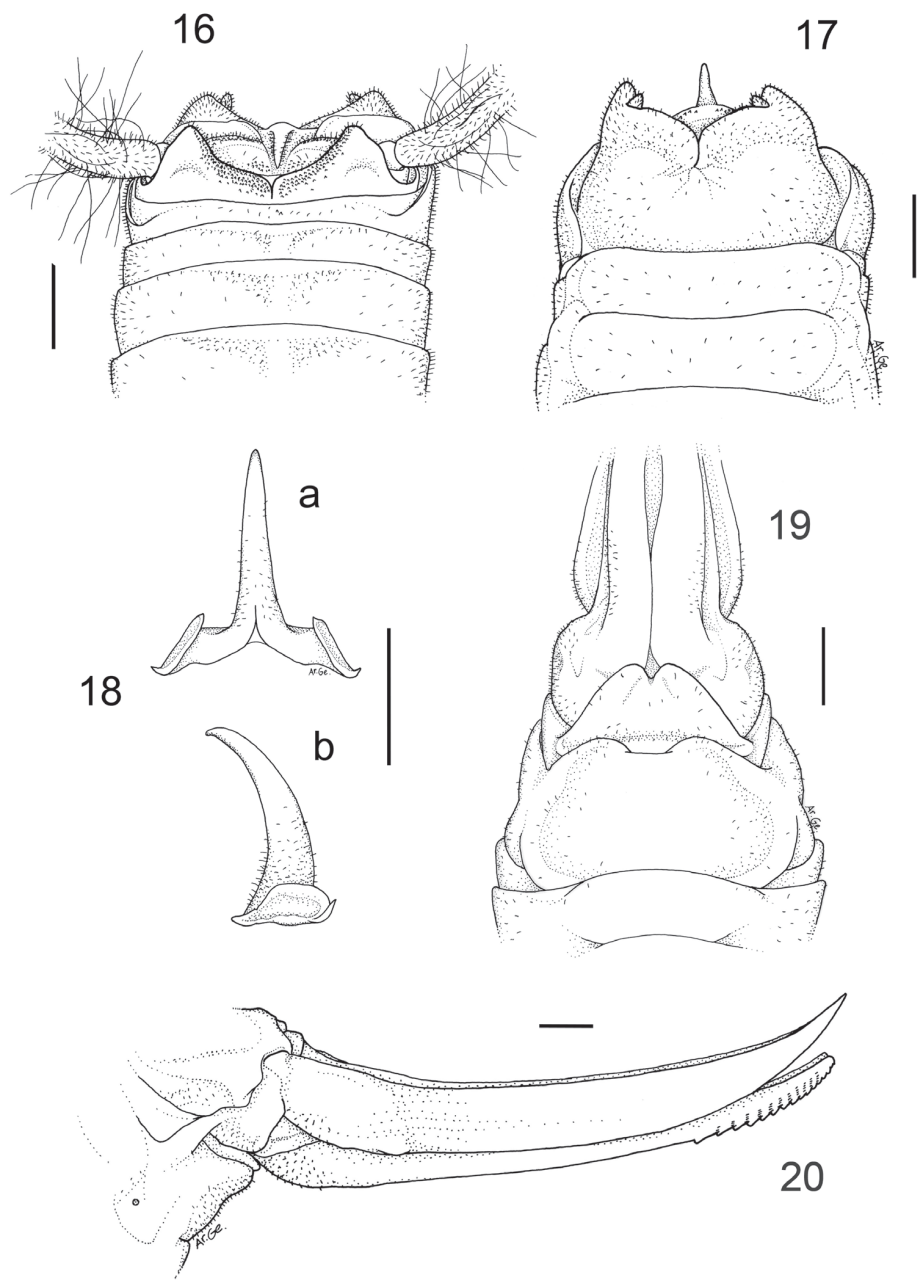
*Dolichopoda calidnae* sp. n. Holotype male **16** X tergite, dorsal view **17** subgenital plate, ventral view **18** epiphallus **a** dorsal view **b** lateral view. Female **19** subgenital plate, ventral view **20** ovipositor, lateral view. Scale bars: 1 mm

**Figure 21.**
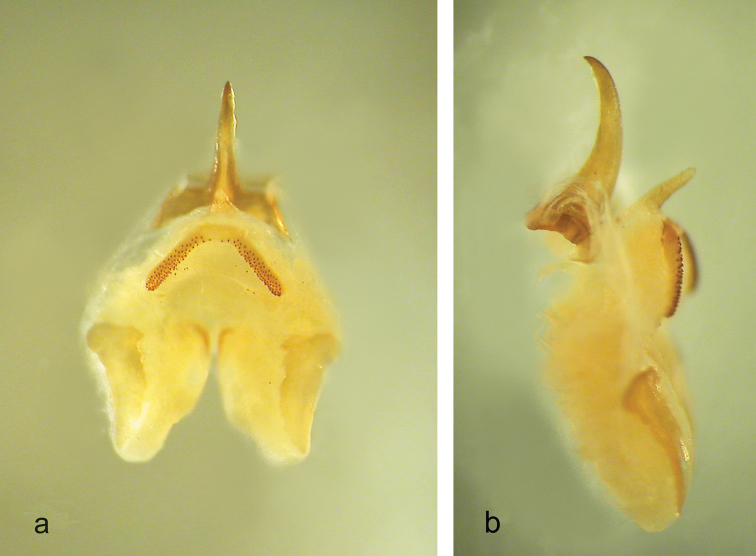
*Dolichopoda calidnae* sp. n., holotype male, epiphallus and accessory apparatus **a** dorsal view **b** lateral view.

Male (holotype). Size rather large. Coloration uniformly pale-testaceus. Legs long and more light in colour. Femora unarmed. Fore tibia armed with 5 spines on both sides of the inferior edge and 3/4 spines on the lower edges. Mid-tibia with 4 short spines on both sides of the upper edge, 4/5 spines on the lower edge. The hind tibia is longer with 21/24 spines of varying length on both sides of the upper edge and 2/3 homogeneous spines on the lower edge. Eighth and ninth abdominal tergites show a sinuous posterior edge, the eighth one is hollower centrally. The tenth tergite, similar to those of the previous species, shows on the posterior edge two large lateral lobes, triangular in shape, with rather rounded apex (Figure 16). Subgenital plate globular at the bottom, with a deep middle incision that runs for half of the total length (Figure 17). Lateral lobes trapezoidal, with two short conical styli. The epiphallus is sclerotized and shows a median process relatively long, almost cylindrical and acute apically. From the side, it appears large at the base and uniformly curved. The basal processes are squared, rather developed and slightly divergent (Figure 18 a, b). The accessory apparatus is similar to that of the previous species (see photo in Figure 21 a, b).

Length (mm): body 17.5; pronotum 3.5; fore femora 14.3; middle femora 14.5; hind femora 22.5; fore tibia 15.9; middle tibia 16.7; hind tibia 26.9; hind tarsus 11.0; 1st article of hind tarsus 5.5.

Female. General appearance as in the male. The length of the body ranges between 16.0–17.0 mm (ovipositor excluded). The subgenital plate is triangular with two moderately incised lobes in the middle (Figure 19). The ovipositor has an average length of 11.0 mm, it is enlarged at the base and regularly curved on the superior edge (Figure 20). The superior valves have a pointed apex and curves upwards, whereas the inferior valves are rounded apically and have 15 denticles.

#### Etymology.

The new species takes its name from the Calidnae Islands (Kalymnos, Leros and Telendos) cited by Homer in Iliad.

## Discussion

**Figure 22.**
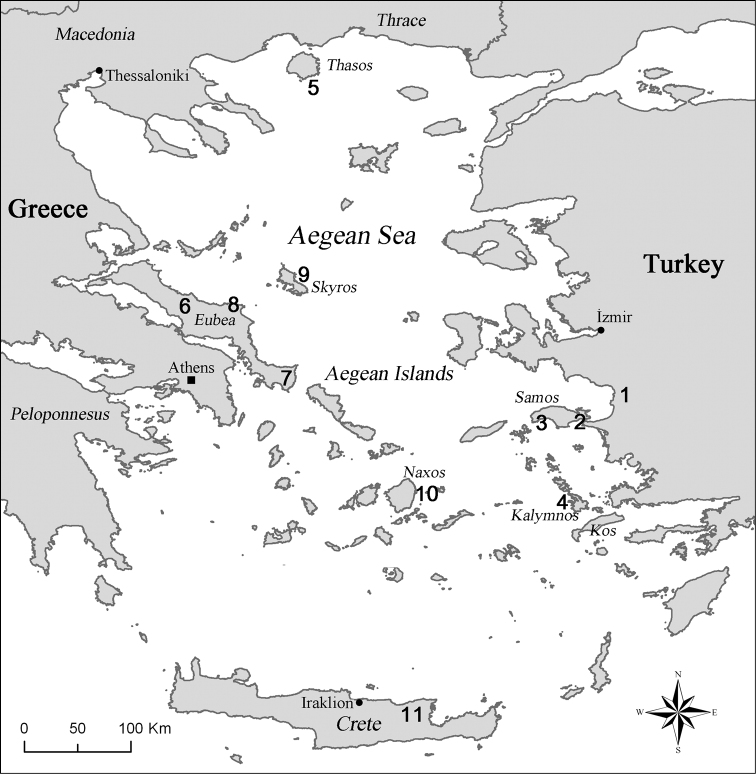
Distribution of troglophilous species of *Dolichopoda* in Aegean Region. **1** *Dolichopoda sutini* sp. n. (Sütini cave, Selçuk, İzmir) **2** *Dolichopoda giulianae* sp. n. (Panaghia Spiliani cave, Pythagorion, Samos) **3** *Dolichopoda kalithea* sp. n. (Kakoperato cave, Kalithea, M. Kerkis, Samos) **4** *Dolichopoda calidnae* sp. n. (Seven Virgins cave, Pothia, Kalymnos) **5** *Dolichopoda thasosensis* (Drakotrypa cave, Thasos) **6** *Dolichopoda makrykapa* (Piyi Nyphi cave, Makrykapa, Eubea) **7** *Dolichopoda cassagnaui* (Aghia Trias cave, Karystos, Eubea) **8** *Dolichopoda ochtoniai* (unnamed cave at North Est of Eubea) **9** *Dolichopoda saraolakosi* (Lynaria caves, Skyros) **10** *Dolichopoda naxia* (Za cave, Filotas, Naxos) **11** *Dolichopoda paraskevi* (Aghia Paraskevi cave, Skotino, Iraklion, Crete).

Here we describe three new species belonging to the cave cricket genus *Dolichopoda* from the Aegean Greek Islands (Southern Sporades) and one from the Western Turkish coast. Considering the additional seven species already reported for the area, there is now a total of 11 recorded species of *Dolichopoda* that currently inhabit caves of the region (Figure 22). These new data, therefore, document the high diversity of the genus in the Hellenic region (25 species in total) reinforcing the hypothesis of a central area of dispersal of *Dolichopoda* corresponding to the ancient Aegean plate (Ruffo 1955).

The new species are all morphologically homogeneous, due to a strong similarity of most of the characters examined (i.e. tenth tergite, epiphallus, accessory apparatus and female subgenital plate). Based on these characters the new species show a clear affinity with *Dolichopoda naxia* from the Cyclades and with *Dolichopoda paraskevi* from Crete (see Table 1). On the other hand, they are well separated from *Dolichopoda thasosensis*, anendemic taxon of the Thasos Island (Tracia), showing a peculiar trilobate shape of the tenth tergite, and from the western species from Eubea and Skyros. These latter species are characterized by a bifurcate apex of the epiphallus and for this reason Boudou-Saltet (1983) placed them in the different sub-genus *Petrochilosina* (*Dolichopoda makrykapa* Boudou-Saltet, 1980, *Dolichopoda ochtoniai* Boudou-Saltet, 1983, *Dolichopoda cassagnaui* Boudou-Saltet, 1971from Eubea and *Dolichopoda saraolakosi* Boudou-Saltet, 1983from Skyros). The sub-genus includes also three additional species (*Dolichopoda insignis* Chopard, 1955, *Dolichopoda petrochilosi* Chopard, 1954 and *Dolichopoda vandeli* Boudou-Saltet, 1970)from Central Greece (Attica and Beotia). Finally, the species *Dolichopoda sutini* from the western Turkish coast doesn’t show any affinity to the other Anatolian species, supporting its indipendent origin from the *Dolichopoda* species living in caves of the Southern Taurus and the Black sea region (Taylan et al. 2011).

The strong morphological homogeneity of this Eastern Aegean species complex, whose range extends now from the Aegean coast of Turkey to the caves of Eastern Crete, clearly suggests a common origin from an ancestor that presumably occupied the eastern part of the ancient Aegean plate. As reported by Allegrucci et al. (2009), the radiation of the genus *Dolichopoda* in this area may have been triggered by the combination of changes in sea level and the relatively humid climate occurring during the Messinian. In that study the origin of the Cretan species *Dolichopoda paraskevi* was estimated at 4.9 million years ago (Mya), shorthly after the end of Messinian salinity crisis (dated at 5.3 Mya). After this period Crete became permanently isolated promoting speciation of *Dolichopoda paraskevi*. The split of *Dolichopoda naxia* (Naxos) from the Eastern Aegean species (Allegrucci et al. 2009), presumably started around 3 Mya. This dating coincides with the separation of the southern Cyclades from the northern Cyclades plateau dated at 3.5 Mya, suggesting that *Dolichopoda naxia* probably represents an old lineage of eastern origin. Finally the affinity of the three new species from the easternmost Aegean islands (Samos and Kalimnos) could be explained in light of the palaeogeography of the area; both islands were in fact connected to continental Asia Minor until recently (i.e. Pleistocene). The age of these species dates back to the end of the Pliocene and the beginning of the Pleistocene, fitting thus with the geological evolution of the islands.

Of particular interest is the situation on Samos Island, where two species (*Dolichopoda kalithea* and *Dolichopoda giulianae*) co-occurr. The differentiation of these two closely related species could be explained by the different geological origin of their cave habitats that could have acted as a geographic and/or ecological barrier preventing gene flow. Kakoperato and Sarandaskaliotissa caves, inhabited by *Dolichopoda kalithea*, open to the Eastern slopes of Mount Kerkis, a massif of dolomitic marble of Cretaceous origin. On the other hand, the Panaghia Spiliani cave, where *Dolichopoda giulianae* lives, is placed in a lacustrine limestone formation of Neogene origin (Ring et al. 1999). The strong affinity between these two species and *Dolichopoda sutini* from Selçuk (Turkey) is justified by the proximity of Samos to the Turkish coast (the island now is separated from the Western Anatolian coast by a channel less than 1.5 Km wide and 30.0 m deep), suggesting that a connection between Samos and the Turkish coast probably existed until the recent Holocene (Stiros et al. 2000, Kayan 2004).

**Table 1.** Mean values of 11 morphological parameters of the Aegean *Dolichopoda* species here studied (dimension in mm).

	*Dolichopoda sutini*	*Dolichopoda calidnae*	*Dolichopoda giulianae*	*Dolichopoda kalithea*	*Dolichopoda naxia*^1^	*Dolichopoda paraskevi*^2^
Body	13.50	18.00	15.00	16.50	19.00	14.5
Pronotum	3.00	3.50	3.50	3.00	4.00	3.00
1 Femora	16.00	14.00	14.00	13.00	16.00	14.5
2 Femora	15.00	15.00	14.00	13.50	16,00	14.00
3 Femora	22.00	23.00	22.00	21.00	25.00	23.50
1 Tibia	17.00	16.00	16.00	15.00	17.00	17.00
2 Tibia	16.50	17.00	17.00	16.00	18.00	17.00
3 Tibia	30.00	30.00	29.00	27.00	33.00	30.50
Hind tarsus	10.00	11.00	11.00	10.00	11.50	10.50
I°art.h.tarsus	5.00	5.50	5.50	5.00	5.50	5.50
Ovipositor	10.00	11.00	11.00	12.00	11.50	11.50

^1^ Boudou-Saltet 1972, ^2^ unpublished data (Rampini and Di Russo)

## Supplementary Material

XML Treatment for Dolichopoda (Dolichopoda) sutini

XML Treatment for Dolichopoda (Dolichopoda) giulianae

XML Treatment for Dolichopoda (Dolichopoda) kalithea

XML Treatment for Dolichopoda (Dolichopoda) calidnae
